# Supplementary material for: 5-fluorocytosine resistance is associated with hypermutation and alterations in capsule biosynthesis in Cryptococcus
Source: Nat Commun. 2020 Jan 8;11:127. doi: 10.1038/s41467-019-13890-z (PMC6949227; doi:10.1038/s41467-019-13890-z)
Supplement: Supplementary file 1 — Supplementary Information [file 41467_2019_13890_MOESM1_ESM.pdf]

# Supplementary Information

# Hypermutation in *Cryptococcus* reveals alterations in capsule biosynthesis are associated with 5-fluorocytosine resistance

R. Blake Billmyre[1†§], Shelly Applen Clancey[1§], Lucy X. Li[2], Tamara L. Doering[2], and Joseph Heitman[1*]

[1]Department of Molecular Genetics and Microbiology, Duke University Medical Center, Durham, NC, USA
[2]Department of Molecular Microbiology, Washington University School of Medicine, Saint Louis, Missouri 63110

**Supplementary Figure 1. VGIIa-like isolates acquire resistance to 5FC and 5FU more rapidly than the VGIIa isolate R265.**

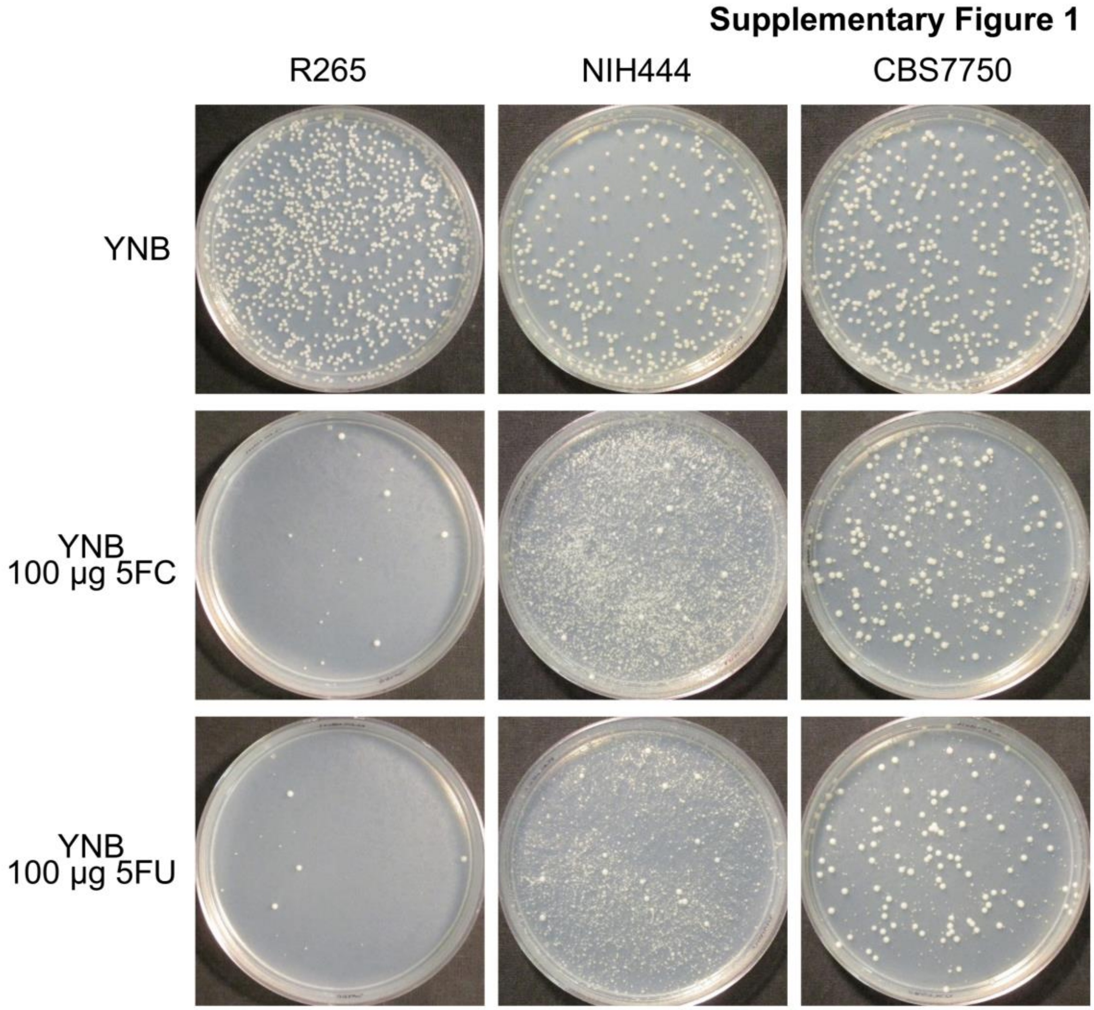


VGIIa-like strains NIH444 and CBS7750 that harbor *msh2* nonsense alleles were tested for the ability to generate resistance to 5FC and 5FU in comparison with the closely related VGIIa strain R265. For each strain, 5 mL YPD cultures were inoculated from a single colony and grown overnight at 30°C. After washing, 100 µl of a $10^{-5}$ dilution was plated to YNB control plates and 100 µl of undiluted cultures was plated on media containing 5FC or 5FU. The VGIIa-like strains generated substantially more isolates resistant to both drugs.

**Supplementary Figure 2. Mutants of *fcy1* and *fur1* in *Cryptococcus neoformans* are resistant to 5FC but not 5FU.**

Supplementary Figure 2

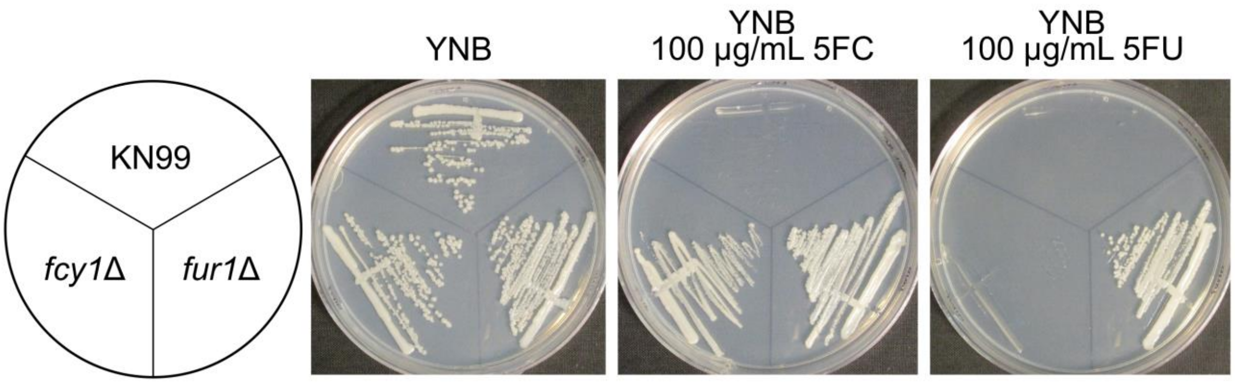


*fur1*Δ and *fcy1*Δ strains from the KN99 *C. neoformans* collection were struck onto YNB, YNB + 100 µg/mL 5FC, and YNB + 100 µg/mL 5FU. While the *fcy1*Δ mutant strain grew on media containing 5FC, it did not grow on media containing 5FU. In contrast, the *fur1*Δ mutant strain grew on media with either drug.

**Supplementary Table 1.** Strains and plasmids used in this study

| Strain name | Genotype | Construction or source |
|---|---|---|
| RBB17 | R265 *MATα msh2Δ::NEO* | Billmyre et al, 2017[1] |
| RBB18 | R265 *MATα msh2Δ::NEO* | Billmyre et al, 2017[1] |
| SEC612 | KN99 *MAT***a** *ugd1Δ::NEO* | Biolistic transformation |
| SEC613 | H99 *MATα ugd1Δ::NEO* | SEC612 x SEC615 |
| SEC614 | KN99 *MAT***a** *uxs1Δ::NAT* | KN99**a** x KN99α *uxs1Δ*::NAT |
| SEC615 | H99/KN99 *MATα uxs1Δ::NAT* | H99 x SEC614 |
| SEC616 | KN99 *MAT***a** *ugd1Δ::NEO uxs1Δ::NAT* | SEC612 x SEC615 |
| SEC617 | H99 *MATα ugd1Δ::NEO uxs1Δ::NAT*-1 | SEC612 x SEC615 |
| SEC618 | H99 *MATα ugd1Δ::NEO uxs1Δ::NAT*-2 | SEC612 x SEC615 |
| TDY1787 | KN99 *MATα uxs1Δ::NAT* | Li et al, 2018[2] |
| TDY1811 | KN99 *MAT***a** *uxs1Δ::NAT UXS1::NEO* | Li et al, 2018[2] |
| TDY1799 | KN99 *MAT***a** $P_{ACT1}$ *UXS1* overexpression (*NAT*) | Gish et al, 2016[3] |
| TDY1679 | KN99 *MAT***a** *uxt1Δ::NEO* | Li et al, 2018[2] |
| TDY1685 | KN99 *MAT***a** *uxt2Δ::NAT* | Li et al, 2018[2] |
| TDY1695 | KN99 *MAT***a** *uxt1Δ::NEO uxt2Δ::NAT* | Li et al, 2018[2] |
| TDY1076 | KN99 *MAT***a** *cxt1Δ::NAT* | Klutts et al, 2008[4] |
| TDY1077 | KN99 *MAT***a** *cxt2Δ::NEO* | Klutts et al, in preparation |
| TDY1078 | KN99 *MAT***a** *cxt1Δ::NAT cxt2Δ::NEO* | Klutts et al, in preparation |
| | KN99 *MATα fur1Δ::NAT* | Madhani collection |
| | KN99 *MATα uxs1Δ::NAT* | Madhani collection |
| | KN99 *MATα fcy1Δ::NAT* | Madhani collection |
| | KN99 *MATα fcy2Δ::NAT* | Madhani collection |

**Supplementary Table 2.** Oligonucleotides used in this study

| Primer | Sequence | Description |
|---|---|---|
| JOHE45233 | gtaacgccagggttttcccagtcacgacgCCAAATGTGTTTGCTATGTG | 5' primer to amplify 1 kb upstream *UGD1* for homologous recombination gene deletion. Includes homology to pGI3. |
| JOHE45085 | ctggccgtcgttttaTTTGAATGGGGTTGAGGGTA | 3' primer to amplify 1 kb upstream *UGD1* for homologous recombination gene deletion. Includes homology to *NEO*. |
| JOHE45086 | TACCCTCAACCCCATTCAAAtaaaacgacggccag | 5' primer to amplify *NEO* for homologous recombination gene deletion of *UGD1*. Includes homology to *UGD1* upstream region. |
| JOHE45087 | GTCGCCGGTACCGATAGTcaggaaacagctatgac | 3' primer to amplify *NEO* for homologous recombination gene deletion of *UGD1*. Includes homology to *UGD1* downstream region. |
| JOHE45088 | gtcatagctgtttcctgACTATCGGTACCGGCGAC | 5' primer to amplify 1 kb downstream *UGD1* for homologous recombination gene deletion. Includes homology to *NEO*. |
| JOHE45234 | gcggataacaatttcacacaggaaacagcCTCACGATTGCCTCATAAAC | 3' primer to amplify 1 kb downstream *UGD1* for homologous recombination gene deletion. Includes homology to pGI3. |
| JOHE45303 | GCGTTGAAGTGGTAAGTG | Internal 5' *UGD1* screening primer |
| JOHE45304 | GACGATCTTGGAAGAGGTAG | Internal 3' *UGD1* screening primer |
| JOHE45335 | GTCCTCGACAACTTCTTCAC | Internal 5' *UXS1* screening primer |
| JOHE45336 | CGGTGATAACCATAGGTC | Internal 3' *UXS1* screening primer |
| JOHE41579 | CTAACTCTACTACACCTCACGGCA | 5' *STE20***a** screening primer |
| JOHE41580 | CGCACTGCAAAATAGATAAGTCTG | 3' *STE20***a** screening primer |
| JOHE41581 | GGCTGCAATCACAGCACCTTAC | 5' *STE20*α screening primer |
| JOHE41582 | CTTCATGACATCACTCCCCTAT | 3' *STE20*α screening primer |

Supplementary References

1. Billmyre, R. B., Clancey, S. A. & Heitman, J. Natural mismatch repair mutations mediate phenotypic diversity and drug resistance in *Cryptococcus deuterogattii*. *eLife* **6**, e28802 (2017).
2. Li, L. X., Rautengarten, C., Heazlewood, J. L. & Doering, T. L. Xylose donor transport is critical for fungal virulence. *PLoS Pathog.* **14**, e1006765 (2018).
3. Gish, S. R. *et al.* Computational analysis reveals a key regulator of cryptococcal virulence and determinant of host response. *mBio* **7**, e00313-16 (2016).
4. Klutts, J. S. & Doering, T. L. Cryptococcal xylosyltransferase 1 (Cxt1p) from *Cryptococcus neoformans* plays a direct role in the synthesis of capsule polysaccharides. *J. Biol. Chem.* **283**, 14327–14334 (2008).
