# Supplementary material for: 5-fluorocytosine resistance is associated with hypermutation and alterations in capsule biosynthesis in Cryptococcus
Source: Nat Commun. 2020 Jan 8;11:127. doi: 10.1038/s41467-019-13890-z (PMC6949227; doi:10.1038/s41467-019-13890-z)
Supplement: Supplementary file 2 — Description of Additional Supplementary Files [file 41467_2019_13890_MOESM2_ESM.pdf]

Description of Additional Supplementary Files

File Name: Supplementary Data 1
Description: **Candidate Polymorphic Variants in Sequenced Strains.** Polymorphic sites identified within the whole genome sequenced strain set with an assigned impact of at least "moderate" via SNPeff. Moderate impact variants are shaded in yellow, while high impact variants were shaded in red.
